# The Health and Life in Balance intervention to improve patient capacity for older people with multimorbidity: a pragmatic mixed methods non-randomised pilot study

**DOI:** 10.1186/s12875-025-02974-z

**Published:** 2025-09-08

**Authors:** Caroline Kappelin, Klas Ytterbrink Nordenskiöld, Elisabeth Bos Sparén, Annica Lagerin, Caroline Wachtler

**Affiliations:** 1 Department of Family Medicine and General Practice, Karolinska Institute, Institution of Neurobiology, Car Sciences, and Society, Alfred Nobel’s Allé 23, Huddinge, 141 83 Sweden; 2 Department of Health Care Sciences, Marie Cederschiöld University, Stigbergsgatan 30, Stockholm, 116 28 Sweden

**Keywords:** Multimorbidity, Interdisciplinary research, Person-centred care, Minimally Disruptive Medicine, Primary care, Feasibility study

## Abstract

**Background:**

The aim of this study was to assess acceptance, feasibility and further need of development of the intervention *Health and Life in Balance (HLB)* for improving patient capacity for older people with multimorbidity.

**Methods:**

A convergent mixed-methods non-randomised pilot study in one intervention (IU) and one control primary care unit (CU) in Region Stockholm, Sweden. General practitioners (GPs) in both units recruited individuals fulfilling eligibility criteria: ≥ 65 years of age, ≥ 2 chronic diseases, and an increased care need. The intervention involved: creating a care plan with a district nurse (DN); DN follow-ups over 6 months; improved communication between DN and GP. The control group received usual care. Quantitative data on preliminary primary, Illness Intrusiveness Rating Scale (IIRS), and secondary outcomes were collected from patient reported outcome measures and medical charts and analysed using statistical analysis. Qualitative data came from patient and healthcare provider interviews and medical charts, analysed using inductive thematic analysis. The mixed-methods analysis used joint display.

**Results:**

Between February and June 2022, 24 and 29 participants were recruited from the intervention and control units respectively. Participants had mean age 79 years and mean number of 18 diagnoses and 10 medications. 56.6% were female. There were no significant differences in preliminary primary (IIRS within group change IU *p* = 1.0, CU *p* = 0.43) or secondary quantitative outcomes. Two themes were identified in the qualitative analysis: *Vulnerable patients need the intervention most* and *Relational continuity and (w)holism are positive and satisfactory but threatened by lack of time and priority.* The joint interpretation identified scheduled holistic nurse follow-ups being acceptable and feasible for individuals in need. However, HLB requires further development to better target individuals with the greatest care needs and to improve the delivery of person-centred care, particularly in terms of aligning with patient priorities and enhancing teamwork.

**Conclusions:**

This mixed-methods pilot study indicates partial acceptance and feasibility of *HLB,* but the intervention should be further developed to target at-need individuals and to raise priority, assess patient-centredness practically and improve teamwork to improve patient centeredness.

**Trial registration:**

The trial was registered in ClinicalTrials.gov on the 24th of January 2025 (clinicaltrials.gov/study/NCT06791135).

**Supplementary Information:**

The online version contains supplementary material available at 10.1186/s12875-025-02974-z.

## Introduction

As the world’s population is getting older, people live longer lives with multiple chronic conditions and needs tailored approaches to address their needs. Individuals with two or more chronic conditions, multimorbidity, are increasing with an aging population [1]. Individuals with multimorbidity are at increased risk of having common mental health problems such as depression [2] and anxiety [3], associated with poor quality of life [4, 5], poor health outcomes [6–9], and great healthcare costs [10, 11]. Moreover, a fragmented primary care system, focusing on treating one disease or problem at a time, many times fail to meet these patients´ needs [12, 13]. Instead, individuals with multimorbidity are at risk of treatment burden involving more healthcare contacts with different healthcare providers and more medications than benefit the individual in the healthcare system today [12, 13]. The World Health Organisation (WHO) have stated a need to develop person-centred and holistic approaches to care, to address patients with multimorbidity to improve their overall health, including their mental health and wellbeing [1]. Moreover, this need has been described in many nations’ policy documents [14–16].

There is a continued need to develop, pilot and implement person-centred interventions in primary care. In recent years, several interventions have been developed and tested involving different aspects of person-centred approaches showing mixed results [17, 18]. Yet there has been some evidence of effectiveness of collaborative care (CC) interventions for individuals with depression and one more chronic disease in decreasing depressive symptoms [17, 19]. Nevertheless, to address individuals with multimorbidity there has been a call to develop interventions not addressing specific diseases and using non-disease specific outcome measures [18]. Hence, there is still a lack of consistency and knowledge on what aspects of care to include, and how care should be provided and evaluated [18].

To address this gap, the intervention *Health and Life in Balance* (HLB) was developed. The intervention development involved a literature review and a qualitative synthesis of CC interventions and included components for individuals with multimorbidity involving common mental health problems [19]. Moreover, it involved a qualitative study of general practitioners’ (GPs) experiences of managing individuals with multimorbidity involving common mental health problems in Swedish primary care [20]. HLB involves an adapted CC design and ICAN discussion aid to address patient capacity and workload. CC involves: Teamwork between a care manger (CM) and a general practitioner (GP); A structured management plan between patient and CM, most often involving medication and CBT in a stepped care model addressing depression treatment; Scheduled patient follow-ups by CM; and Improved interprofessional communication. Improved interprofessional communication can involve for GPs and DNs having appointments to discuss patients scheduled or ad hoc [21]. ICAN discussion aid is based on the principles of Minimally Disruptive Medicine (MDM). MDM is a person-centred approach based on the cumulative complexity model for individuals with multimorbidity [22]. This approach and model identify individuals with multimorbidity to be at risk of an imbalance between decreased capacity due to disabling symptoms, and increased workload due to many visits to different healthcare providers prescribing many medications and giving several recommendations [12, 22]. This imbalance increases the risk of burden of illness and treatment respectively [12]. ICAN discussion aid is a written tool for individuals with at least one chronic disease to fill out before seeing a healthcare provider [23]. Then the content of the filled out ICAN is discussed between the individual and the healthcare provider in a meeting aiming to facilitate clinical conversations. The tool addresses five aspects that can enhance or lower patient capacity: the patient’s ability to adapt to new life circumstances; patient resources such as physical energy, time and health literacy; patient social network and ability; doing the work of being a patient; and support provided by the healthcare environment [24]. The ICAN discussion aid has been shown to be feasible for individuals with at least one chronic condition in an American context [23]. In addition, MDM-informed interventions have been associated with reduced readmissions to hospitals for patients with multimorbidity [25].

The purpose of this convergent mixed methods pilot study was to assess the acceptance, feasibility and further need of development of the HLB intervention for improving patient capacity for older individuals with multimorbidity.

## Methods

The CONSORT guidelines for pilot studies was used [26] when reporting the study. In addition, the Mixed-methods appraisal tool [27] was used to guide reporting of the mixed-methods design.

### Study design and objectives

A pragmatic mixed methods non-randomised pilot study design was applied to understand feasibility and acceptability and further need of development of the HLB intervention. The study design involved to collect and analyse quantitative and qualitative data in parallel and then merging the two analyses together interpreting how the two data analyses converge and diverge using a joint display. The rationale for this mixed methods approach was that quantitative data provides an overview of participants characteristics, intervention components delivered, and potential effect of the intervention, while qualitative data can give insight into the experiences of participants and healthcare providers of the intervention. Merging the two types of data analyses together in a joint interpretation can lead to a more comprehensive understanding of how to optimise the HLB intervention for a future effectiveness trial.

The study involved the following quantitative (QUAN), qualitative (QUAL) and mixed methods (MIXED) research questions:

1. What were the characteristics of participants recruited to the pilot study? (QUAN),
2. What components were delivered in HLB? (QUAN)
3. Was HLB associated with improvements in the proposed primary and secondary outcomes? (QUAN)
4. How did participants and healthcare providers experience HLB regarding participant selection, intervention content and impact on health? (QUAL)
5. What participants should HLB target and how should HLB be delivered in a future trial? (MIXED)

### Recruitment and setting

Because of known problems with contamination when testing complex interventions in primary care, a non-randomized clustered intervention pilot design was chosen. Two primary care units were purposively invited, one to be an intervention unit and one to be a control unit. The practices were about the same size and situated in similar socioeconomic suburban areas outside of Stockholm, Sweden. The heads in both units were approached and both units accepted. In the intervention unit, the head of the unit recruited three GPs having the oldest individuals in their patient lists. Moreover, the head recruited three DNs who already worked closely with the recruited GPs. All GPs and DNs accepted.

The research team planned to recruit 30 participants in each unit. This number was considered to be a reasonable number of participants to include during the limited period for inclusion. Eligibility criteria were age ≥ 65 years, having ≥ 2 chronic diseases, and an increased care need determined by the individual’s GP. The age criteria was chosen as multimorbidity is more prevalent with higher age [1] and as previous RCTs of person-centred interventions for individuals with multimorbidity most commonly have recruited older individuals [18]. To address individuals with multimorbidity and an increased care need, there is no shared consensus in which individuals to include and previous studies have used different inclusion criteria [18]. The research team decided to use the definition of multimorbidity, having ≥ 2 chronic diseases [1], and let the recruiting GPs identify individuals with an increased care need. Exclusion criteria were: Receiving home healthcare; On-going treatment in a secondary unit making individuals unable to utilize the intervention, for example ongoing oncological treatment; or cognitive impairment restricting ability to give informed consent.

Initially, the three GPs participating in the intervention and all GPs in the control unit identified and invited eligible individuals to the study. However, due to poor inclusion rate in the intervention unit, recruitment strategy was changed so the three intervention DNs could also invite individuals in agreement with the GP. Eligible individuals were contacted by a research nurse by telephone. Interested individuals met with the research nurse and provided written informed consent. In addition, the research nurse wrote digital reminders to not forget to include individuals to the participating GPs in the intervention unit a couple of times during the period of inclusion.

### Intervention

HLB was a 6-month long intervention and involved:

1. Three specified teams of one DN and one GP in each team
2. The participant met with the DN in 1–2 visits during the 2 first weeks of the intervention to:
  Go through ICAN discussion aid divided into three parts addressing areas of reduced capacity, increased workload, and three open questions of what is most important to the individual. See Additional file 1 [23].
  Set up a care plan defining goals from ICAN discussion aid to work with during the intervention.
  Plan scheduled follow-ups.
3. Scheduled follow-ups:
  DN and the participant decided on number, types and content of follow-up appointments adjusted to the participant’s needs, preferences and care plan.
  One final visit after 6 months to follow-up and evaluate the care plan and achievements from the intervention.
    ◦ The research nurse wrote a digital reminder to the intervention nurses when it was time to book a final visit.
4. Enhanced interprofessional communication
  Improved communication between DN and GP about included participants according to team preferences. This could involve booking a meeting to discuss intervention participants.

**Fig. 1:**
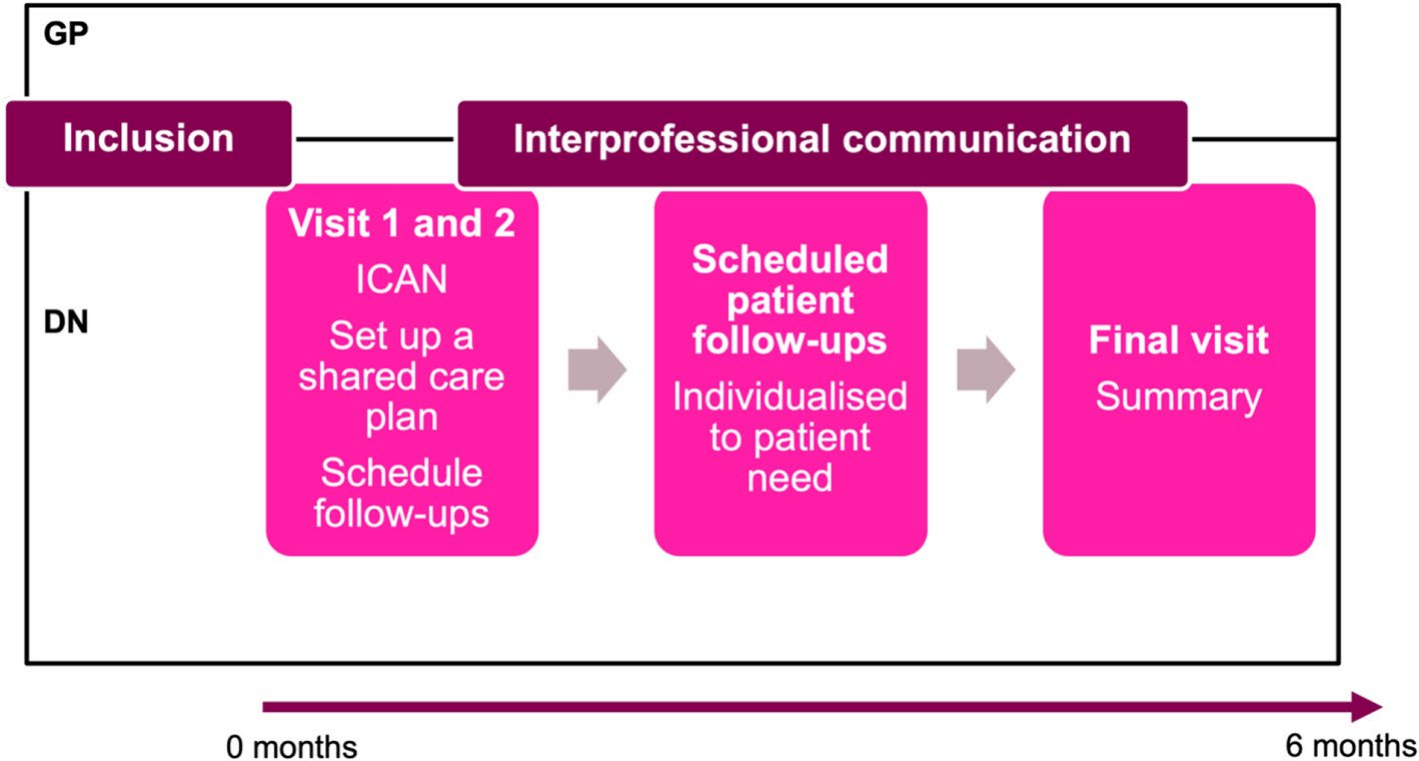
The Health and Life in Balance intervention. A visual presentation of intervention components and delivery for 6 months

HLB differs from usual care in Swedish primary care regarding to provide point 2 and 3. Regarding existing structures for teamwork, point 1, and improved communication, point 4, they can vary between different primary care units in the existing healthcare system. This can involve formal or informal existing teams of one GP and one DN working together with communication scheduled or ad hoc in some primary care units, as in this intervention. It can also involve teamwork between all or some GPs and DNs but without specific smaller teams, with or without structured ways for communication in other primary care units (Fig. 1).

#### Pre-intervention information and training

Before the intervention started, intervention GPs and DNs participated in three co-production meetings with the research team to discuss how to practically carry out the intervention. This involved to go through inclusion criteria and discuss how to recruit eligible participants and how to pass them on to the research nurse for further information and to fill out informed consent. Furthermore, it involved how to practically improve interprofessional communication. The teams did not want to have planned meetings to discuss intervention participants. Instead, they wanted to discuss participants when needed. Moreover, the DNs were offered supervision or to have planned meetings with the research nurse to discuss potential difficulties during the intervention. However, they declined.

Finally, GPs and DNs participated in a joint one-hour information meeting about the final intervention design, the intervention material, and about inclusion and team-communication with the research team. Then the DNs had one more hour of information and training in how to use ICAN discussion aid, and how and where to write a care plan.

GPs in the control unit had one information meeting on how to include participants, with the same information about inclusion criteria and how to pass on eligible individuals to meet with the research nurse as in the intervention unit.

### Control primary care unit

Participants in the control unit received care as usual, which in Region Stockholm usually includes annual visits to the GP for individuals with chronic somatic diseases. Otherwise, individuals contact the primary care unit when in need of care.

### Data collection

#### Quantitative data

Baseline data including age, sex, and number and types of diagnoses and medications were collected from patient medical records in both intervention and control unit after the intervention was completed. Descriptive outcomes of how the intervention was delivered included number and types of contacts with intervention DN, care plan documentation, ICAN discussion aid, final visit, communication between GP and DN, and supervision—were also gathered from patient medical records in the intervention unit after the intervention concluded. Primary and secondary outcomes were assessed at baseline and post-intervention in both units. All patient reported outcome measurements (PROMs) were filled out electronically at the same time with assistance of a research nurse. Additional data were collected from patient medical records, including the number of medications, visits to primary and secondary care, and hospital admissions.

##### Outcome measurements in a future randomised controlled trial

The Illness Intrusiveness Rating Scale (IIRS) was selected as the primary outcome measure and analysed for within group difference at 0 and 6 months. The IIRS is a PROM consisting of 13 items, each rated on a 7-point scale, yielding a total score between 13 and 91, along with three subdomain scores. Lower values indicate lower illness intrusiveness [28, 29]. Two types of measurements were used for secondary outcomes. The first type involved PROMs, analysed for within-group differences at 0 and 6 months: Treatment burden— [30]; Quality of Life—EQ-5D-5L—EuroQol [31]; Depressive symptoms— [32]; Worry— [33]; General health—WHODAS 2.0—WHO Disability Assessment Schedule [34]; Alcohol consumption—AUDIT—The Alcohol Use Disorders Identification Test, 12-item version [35]; Drug consumption—DUDIT – The Drug Use Disorders Identification Test [36]. The second type of measurement involved clinical outcomes, analysed for between-group differences at 0 and 6 months: Number of medications; Number of visits to primary care (excluding the intervention); Number of visits to secondary care; and Number of hospital admissions. For a full description of primary and secondary outcomes measures, and their comparison method, see Additional file 2. The choice of several PROMs as primary and secondary outcomes regards a lack of full consensus in which PROM to use when evaluating complex interventions for individuals with multimorbidity and a will to evaluate the use of both ICAN discussion aid and CC components. To date, quality of life, EQ-5D-5L, has been the most frequently used PROM and is the suggested PROM to use in complex interventions for individuals with multimorbidity [18]. However, in previous intervention studies for individuals with multimorbidity, EQ-5D-5L has shown lack of change over time [18], why it was chosen to be secondary outcome in this study. Instead, IIRS was chosen as primary outcome as it aligns with the theory of MDM and ICAN discussion aid, addressing symptom burden [12, 22, 23]. Furthermore, MTBQ reflect the other aspect of MDM and ICAN discussion aid being treatment burden. Regarding to include PROMs addressing aspects of mental health, this regards individuals with multimorbidity being at risk of common mental health problems [2, 3], and the use of CC components in HLB [20, 34]. In a future trial the aim was to use fewer PROMs based on the findings from the pilot study.

##### Changes to pilot trial measurements after the pilot trial commenced

Initially we planned to have two PROMs to evaluate depressive symptoms, both PHQ-9 and Montgomery-Åsberg Depression Rating Scale (MADRS). However, after enrolling the first two participants in the intervention unit we removed MADRS because participants complained of redundancy.

#### Qualitative data

All qualitative data were collected from the intervention unit. All authors participated in developing a semi-structured interview guide for individual participant interviews and focus group interviews with GPs and DNs, see Additional file 3. All participants at the intervention unit were invited to an interview at the final intervention visit with the research nurse. 14 participants accepted the invitation and KYN conducted 14 individual interviews, lasting from 30–60 min, between October 2022 and January 2023 in the participants’ homes [6], by telephone [7] and in the primary care unit [1]. CW observed one interview. AL and EBS conducted two focus group interviews with the three participating DNs in the intervention unit, one in June and one in December 2022. However, as one DN could not participate in December, they conducted an individual interview with her in January 2023. Furthermore, AL and EBS conducted one focus group interview with the three participating GPs in January 2023. All interviews were audio recorded and transcribed by a transcription company. CK and CW took field notes during 3 h-long lunch meetings with the intervention teams during the intervention, in March, June and August 2022. Qualitative data were also collected from all participants in the intervention unit’s medical charts from the intervention period. CK and KYN read the participants’ medical charts for the intervention period searching for potential written data addressing the qualitative research question.

Regarding sample size, the research team was limited to the participating healthcare providers and participants in the intervention unit. However, the sample size was deemed enough to gain information power having a narrow aim, a dense specificity, a partly applied theory, a medium strong dialogue and a case analysis strategy [35].

### Data analysis

#### Quantitative data

Outcomes and participant age distributions were assessed for approximate normality using histograms. Diagnoses and medications were grouped and presented as percentages of the total based on their closest affiliation in the ICD and ATC systems respectively. Baseline differences in gender distribution between treatment groups were assessed using Fisher’s exact test, while differences in age, number of diagnoses, and number of medications were examined using unpaired, two-tailed, t-tests. F-tests were conducted to verify that there were no significant differences in variance between the groups.

To describe the components of HLB delivery, Excel was used to summarize component counts, as well as calculate means and percentages.

Given our small sample size, non-randomized recruitment, and the early identification of substantial missing data in PROMs, we opted for paired sample, two-tailed t-tests to analyse within-group differences pre- and post-intervention, stratified based on intervention status. This approach allowed us to control for individual variability and for the possible detection of unanticipated changes in the control group. Unpaired sample t-tests assessed changes in medication count, primary and secondary care contacts, and hospital admissions between treatment groups. Imputation techniques for missing questionnaire data were not used, as the study aimed to evaluate feasibility rather than estimate effect sizes. Questionnaire completion rates were examined by treating all questionnaires with missing values as incomplete, with counts and percentages summarized in Excel. Analyses followed an intention-to-treat approach. STATA v14.2 was used for statistical analysis. Acceptability of questionnaires was estimated by completion rate: only fully entered questionnaires at baseline and post-treatment were considered complete.

#### Qualitative data

Reflexive thematic analysis with an inductive approach by Braun and Clarke [36] was used for the qualitative analysis. The COREQ guidelines [37] and Braun and Clarke checklist were used for reporting reflexive thematic analysis [38]. An inductive approach was chosen because it allowed to interpret data exploratively, making room for new ideas and interpretations to healthcare providers’ and participants’ experiences of HLB. The authors are all researchers with experiences of qualitative analysis working in primary care as GPs (CW, CK), resident physician (KYN), and DNs (EBS, AL). Two of the authors developed the intervention (CW, CK) and the others conducted the qualitative interviews (KYN, EBS, AL). All authors read the transcripts. CK and KYN coded all data. CK arranged codes into term categories for the first grouping. All authors read the categories and together they participated in an iterative process of generating analytical subthemes. CK checked that the analytical subthemes were in line with relevant data. All authors discussed how the analytical subthemes were related and together developed two themes. CK wrote the first draft of the qualitative result and identified citations to reflect the themes and subthemes. CK translated citations from Swedish to English. All authors read the manuscript and gave feedback and CK further revised it. CK managed data in Excel during the qualitative analysis.

#### Joint display to analyse quantitative and qualitative data on interpretation level

A joint display mixed methods integrated analysis was chosen to answer the main mixed-methods question using an integrated results matrix, involving three columns. In the first two columns an overview of the results from the quantitative and qualitative analyses was presented respectively. Then CK, KYN and CW met, discussed and interpreted how quantitative and qualitative analyses deepened and broadened the understanding to answer the mixed methods question. In the third column, the interpretation of both quantitative and qualitative data analyses together was presented.

## Results

### Recruitment and inclusion

**Fig. 2:**
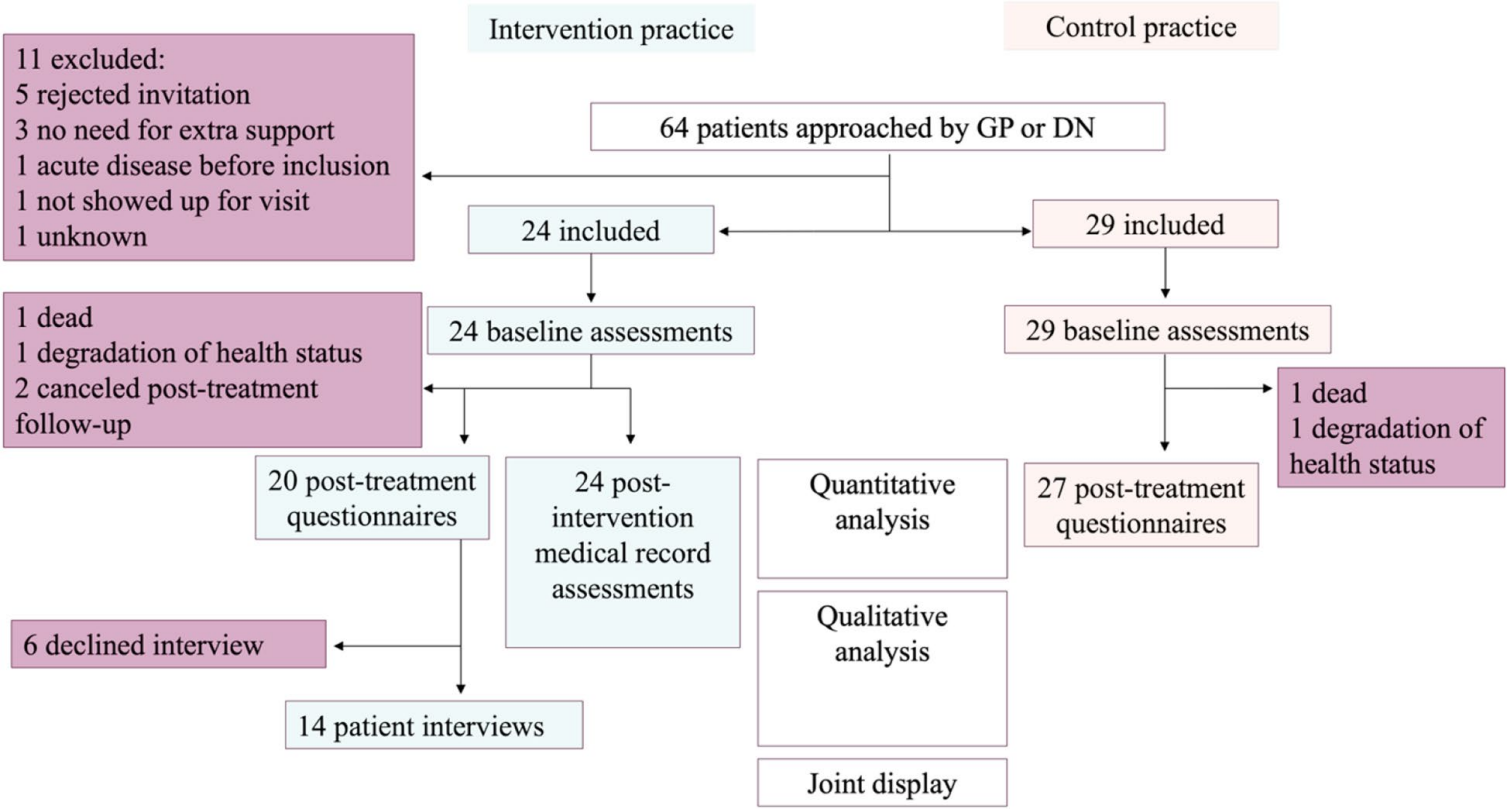
Recruitment, data-collection and analysis flowchart

Recruitment took place between February and June 2022. 24 participants were included to the intervention group and 29 to the control group. Loss to follow-up concerned 6 participants, 4 in the intervention group and 2 in the control group. All participants underwent post-treatment medical record assessment. For a full detail description of *Recruitment, data-collection and analysis flowchart, see* (Fig. 2).

### Quantitative results

#### Baseline data

Participants had a mean age of 78.8 years, and 56.6% were female. On average, they had 17.9 diagnoses, and 10.1 medications (Table 1).

**Table 1:** Baseline demographics and medical aspects by intervention status

**Demographics**				
	Overall (*N* = 53)	HLB (*N* = 24)	TAU (*N* = 29)	*p*-value
Age M (min–max)	78.8(66–93)	80.4 (66–93)	77.4 (66–89)	*p* = 0.12
Female gender, n (%)	30 (56.6%)	14 (58%)	16 (55%)	*p* = 0.52
**Medical aspects**				
	Overall (*N* = 53)	HLB (*N* = 24)	TAU (*N* = 29)	*p*-value
Number of diagnoses, M (min–max)	17.9 (5–40)	19.4 (9–40)	16.6 (5–38)	*p* = 0.18
Number of medications M (min–max)	10.1 (2–22)	10.6 (6–22)	9.7 (2–21)	*p* = 0.49
	HLB (*N* = 24)		TAU (*N* = 29)	
10 most common conditions (%)	Hypertension	76%	Hypertension	79%
	Pain in limb, joint, muscle, abdomen or back	69%	Pain in limb, joint, muscle, abdomen or back	79%
	Arthrosis	41%	Arthrosis	67%
	Diabetes mellitus type 2	34%	Atrial fibrillation and flutter	29%
	Acute upper respiratory infection	21%	Renal failure	29%
	Degeneration of macula and posterior pole	21%	Sleep disturbance	29%
	Dizziness and giddiness	21%	Hypothyroidism	25%
	Local infection of skin, unspecified	17%	Degeneration of macula and posterior pole	21%
	Malaise and fatigue	17%	Diabetes mellitus type 2	21%
	Sleep disturbance	17%	Dizziness and giddiness	17%
10 most common medications (%)	Antihypertensives	76%	Antihypertensives	88%
	Statins	59%	Statins	67%
	Emollients and Protectives	41%	Paracetamol	58%
	Laxatives	31%	Proton Pump Inhibitors	46%
	Paracetamol	31%	Benzodiazepine Related Drugs	42%
	Desloratadine	28%	Laxatives	29%
	Proton Pump Inhibitors	24%	Levothyroxine	29%
	Benzodiazepine Related Drugs	24%	Apixaban	21%
	Antiinflammatory Drugs, Non-Steroids	21%	Cyanocobalamin	21%
	Cyanocobalamin	21%	Furosemide	21%

*HLB* Health and life in balance, *TAU* Treatment as usual, *M* Mean

#### Intervention delivery

**Fig. 3:**
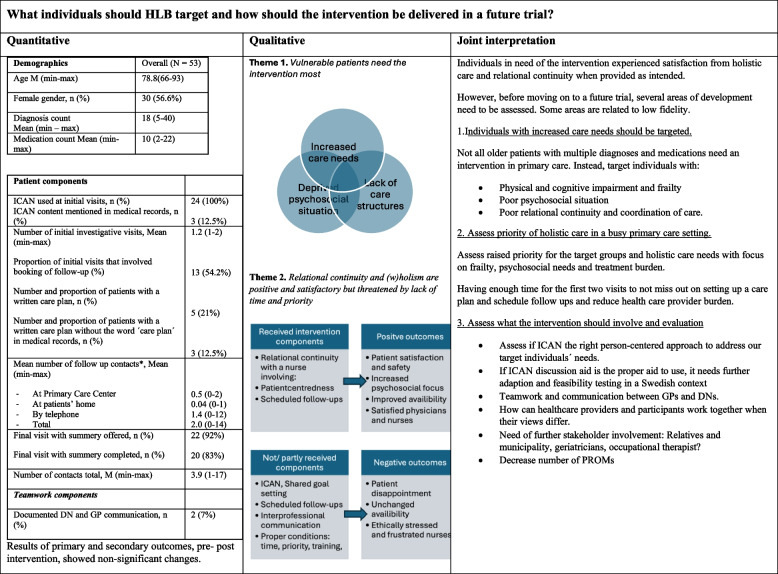
Integrated results matrix *Joint interpretation of the mixed methods research question.* What patients should HLB target and how should the intervention be delivered in a future trial?

Participants received an average of 1.2 initial visits with a DN. ICAN discussion aid was filled out by all (100%) of the participants and 21% had a documented care-plan. A follow-up was scheduled for 54.2% for the participants in the initial visit. Mean number of follow-up contacts were 2, where 1.4 were done by telephone. 92% of the participants were offered, and 83% had a final visit. There were two instances of documented communication between a DN and GP during the intervention (Fig. 3).

#### Primary and secondary outcomes

No significant effects on primary or secondary outcome measures were observed (Table 2). The percentage of PROMs that were fully entered and correctly filled out ranged from 28% for IIRS to 83% for EQ-5D-5L. For a full summery of the completion rates, see Additional file 3.

**Table 2:** Primary and secondary outcomes, at Baseline and Posttreatment, presented with Within group change or Between group difference

	*Baseline M (min–max)*		*Posttreatment (6 months)*		Within group change	
	*HLB*	*TAU*	*HLB*	*TAU*	HLB	TAU
**Primary outcome**						
** IIRS**						
Total score	47.3 (24–65) *n* = 3	33.9 (15–67) *n* = 12	47.0 (23–59) *n* = 3	32.0 (14–79) *n* = 12	*p* = 1.00	*p* = 0.43
Domains						
* - Relationship*	*17.8 (6–31) n* = *5*	*12.5 (6–26) n* = *15*	*17.8 (9–28) n* = *5*	*11.2 (6–33) n* = *15*	*p* = *1.00*	*p* = *0.31*
* -Intimacy*	*5.3 (2–12) n* = *6*	*5.6 (2–13) n* = *17*	*6.3 (2–12) n* = *6*	*5.9 (2–14) n* = *17*	*p* = *0.43*	*p* = *0.77*
* - Instrumental*	*17.9 (11–26) n* = *11*	*13.9 (5–28) n* = *18*	*16.1 (5–25) n* = *11*	*14.9 (5–32) n* = *18*	*p* = *0.83*	*p* = *0.57*
**Secondary outcomes**						
* Patient reported outcome measures*						
MTBQ	34.9 (24–43) *n* = 17	36.6 (19–50) *n* = 22	34.7 (25–41) *n* = 17	37.2 (18–49) *n* = 22	*p* = 0.92	*p* = 0.52
EQ-5D-5L	0.66 (0.2–0.94) *n* = 19	0.77 (0.28–0.94) *n* = 25	0.68 (0.28–0.94) *n* = 19	0.77 (0.23–1.0) *n* = 25	*p* = 0.68	*p* = 0.96
PHQ-9	5.5 (1–10) *n* = 13	3.5 (0–15) *n* = 22	5.3 (0–15) *n* = 13	4.4 (0–24) *n* = 22	*p* = 0.93	*p* = 0.19
PSWQ	39.2 (28–53) *n* = 12	35.1 (20–48) *n* = 21	39.8 (32–52) *n* = 12	38.3 (23–69) *n* = 21	*p* = 0.79	*p* = 0.22
WHODAS	23.9 (13–30) *n* = 11	19.5 (13–35) *n* = 19	22.0 (12–36) *n* = 11	19.7 (12–38) *n* = 19	*p* = 0.22	*p* = 0.77
AUDIT	3.8 (2–7) *n* = 10	2.9 (1–10) *n* = 20	4.1 (2–9) *n* = 10	2.8 (0–11) *n* = 20	*p* = 0.47	*p* = 0.58
DUDIT	(0–0) *n* = 10	0.18 (0–3) *n* = 22	0.0 (0–0) *n* = 10	0.23 (0–5) *n* = 22	-	*p* = 0.67
* Medical aspects*						
Number of medications	10.6 (6–22) *n* = 24	9.7 (2–21) *n* = 29	10.9 (7–23) *n* = 23	9.9 (3–24) *n* = 28	*p* = 0.72	*p* = 0.86
			HLB	TAU	Between group difference	
Number of primary care contacts^1^			12.0 (2–63) *n* = 24	9.4 (1–33) *n* = 29	*p* = 0.43	
Number of secondary care contacts^2^			2.7 (0–15) *n* = 24	3.9 (0–13) *n* = 29	*p* = 0.26	
Number of hospital admissions^2^			0.2 (0–2) *n* = 24	0.1 (0–2) *n* = 29	*p* = 0.59	

*HLB* Health and life in balance, *TAU* Treatment as usual, *IIRS* Illness Intrusiveness Ratings Scale, *MTBQ* Multimorbidity Treatment Burden Questionnaire, *EQ5D5L* The EuroQol 5D, *PHQ-9* The Patient Health Questionnaire, *PSWQ* The Penn State Worry Questionnaire, *WHODAS* WHO Disability Assessment Schedule, *AUDIT* the Alcohol Use Disorders Identification Test, *DUDIT* the Drug Use Disorders Identification Test

^1^Outside the intervention, during the intervention time-period

^2^During the intervention time-period

### Qualitative results

We generated two themes in our qualitative analysis: *Vulnerable patients need the intervention most* and *Relational continuity and (w)holism are positive and satisfactory but threatened by lack of time and priority.* The two themes interrelate, as when the intervention was matched to the very vulnerable participants who needed it the most, it generated patient and healthcare provider satisfaction and safety. However, mismatch between patients and intervention delivery involved risk of potential patient harm and healthcare provider stress.

#### Theme 1. Vulnerable patients need the intervention most

Patients and healthcare providers described vulnerable patients as those in most need of the intervention. Vulnerability was seen as reflecting individual and systemic aspects including increased care needs because of frailty and aging, deprived psychosocial situation including poor coping strategies, and lack of ongoing relational continuity, holistic thinking, and coordination of care for patients with many diseases and/or healthcare providers. However, in this study some less vulnerable patients were included without a need.

*“Patients who need a look through of their medical questions but also things like loneliness, network, how to contact the municipality. Those are the patients in need of it (the intervention). DN 3*

##### Frailty and destabilizing events lead to increased care needs

Patients and healthcare providers felt that vulnerable patients, with physical and cognitive symptoms related to frailty and risk of isolation and poor quality of life, had most need of the intervention. In particular, physical and cognitive impairment due to aging led to vulnerability and frailty followed by lowered functional level.

*“Yes, this thing that I might have worse memory than my son has. But it’s also something related to my age. I have discovered that some things have got lost from my memory, that I definitively wouldn’t have forgotten before. I am not really confused or anything, or demented. But my memory is clearly worse than five or ten years ago.” Patient, 20*

Sudden life threating events were understood to increase vulnerability and care needs, including the patients’ experience of safety.

*“Actually, I almost died from pneumonia in 2008 and during my hospital admission I had heart fibrillation. Since then, I have had 20–25 electrical cardioversions, I have made two ablations, and I have done a balloon dilatation, and had a couple of pneumonias more. And then I have had these problems with my heart time and again. And it is a pretty big thing, and it’s pretty… In a way…. I would wish for more time with the doctor than exists today, to get answers to all my questions.” Patient, 34*

Patients who felt physically and mentally well, regardless of age, expressed less need of the intervention.

*“I didn’t feel that it concerned me really. Even though I am retired, I felt it didn’t concern me because I don’t need so much care” Patient, 23*

##### Patients with deprived psychosocial situation and lack of coping strategies are seen as in need, but patients do not always want help

Patient and healthcare providers described patients with a deprived psychosocial situation as having increased vulnerability and need for the intervention. A deprived psychosocial situation could involve loneliness, living alone, and lacking psychosocial support. It could also involve worry about health, family and friends, or economic situation and crises throughout life such as losing a partner.

*“I have had a big prosthesis in my left shoulder. And then I have had a lot of troubles with my son, because he is bipolar. It has been difficult with him for quite some time really. And then I talked to the nurse, it was almost like a psychological talk, and that was good. But I do have easy to talk with some friends too. But it was, it felt good to talk with her absolutely.” Patient, 34*

Healthcare providers highlighted that poor coping strategies negatively affect patients’ psychosocial situation, leading to patient seeking care more often than medically needed, and increasing their need for the intervention.

*“The ones who need, those who seek care often and for the same cause all the time. That you get a feeling of security if you know that the nurse calls you once a week or that you know that you have something.” GP 2*

In addition, DNs pointed out that patients with medical risk not known to providers would have most benefit from the intervention but are hard to identify.

*“In my opinion, it is people who are lonelier, maybe with difficulties expressing what they want and need. It is those patients. It takes a little while to find them, the first meeting might be a bit shallower, and then you meet them several times and get a little closer and they dare to open up more. There is where our biggest lack is in missing out on relational continuity is, I believe.” DN 1*

On the other hand, there was a mismatch between what patients with poor psychosocial situation and coping strategies needed and what they wanted. This involved poor motivation to make changes in and adjust to events through life. Healthcare providers experienced frustration and ethical stress when they identified a need and offered care and patients declined, including when patients declined participation. Yet, when patients declined help from a DN, they expressed they wanted to see a GP for medical help, rather than seeing a DN to address psychosocial needs they did not experience themselves.

*“So far, I don’t need any contact with the district nurses and encroach on their time. But I probably will visit the physician again, but then as I said out of medical reasons, not out of social-medical ones”. Patient, 31*

*“The ones who accepted, were the healthy and fit ones knowing how to do everything. It was nice, but it can be a waste of time and energy as it’s not the target group in need of the intervention. Instead, it is the other target group (we want to include), who declined. I experienced it in the same way in this study, the ones who accepted had been included in previous studies or had something similar.” DN 1*

Many of the included patients had a supportive psychosocial situation with help from family and friends, good economy and good coping strategies with less worry and need of the intervention.

##### Vulnerability increases in a fragmented healthcare system

Patients with increased care needs who lacked relational continuity could feel insecure and vulnerable in today’s healthcare system. Lack of relational continuity involved lack of holistic perspective, lack of availability of care other than for acute symptoms and strictly medical needs, and lack of coordination of care.

*“Yes, it feels like you need to be seriously ill, to contact the primary care centre. To have more acute illnesses so to speak.” Patient, 26*

In particular, patients with many different healthcare providers were seen as particularly unsafe in today’s fragmented healthcare system both within the primary care unit as well as in different care units.

*“As you hear, it’s a bit difficult to keep all my care units in order and it (the care) needs to be coordinated. It is the problem that, you have to keep track of all these physicians when you have different care units all by yourself so to speak.” Patient, 33*

*“I think it’s quite a lot about accessibility to healthcare—it comes up at every visit—how to reach different services and whether one needs help getting in touch with other healthcare providers, physiotherapists, occupational therapists, and so on.”. DN 3*

Individuals with functioning relational continuity and follow-ups, such as for individuals with diabetes, described feeling safe and satisfied with their existing care and expressed no need for further intervention content after the first visit.

*“Thanks to my GP who has shown me so much consideration and called to see how I am. Every time I have been to surgery, he has always called and checked on me afterwards. That is something I appreciate.” Patient, 28*

#### Theme 2. Relational continuity and (w)holism are positive and satisfactory but threatened by lack of time and priority

Both patients and healthcare providers felt safe and satisfied when individuals with increased care needs received relational continuity to a nurse, care tailored for their needs, and flexibility including scheduled follow ups. However, some included patients did not receive the intervention apart from a first and final visit. Some of those patients did not experience a need. Others, experiencing a need but without feeling they received any further intervention content, were left with feelings of disappointment. DNs expressed ethical stress to address non-medical needs and an increased workload making them unable to properly provide the intervention.

##### Relational continuity, flexibility, and holistic perspective improved care delivery and made patients feel safe

Both patients and healthcare providers had positive experiences of the intervention including improved relational continuity, flexibility, and holistic thinking. Patients who received the intervention described feeling safe because visits addressed other problems than strictly medical ones, focused on psychosocial situation and provided support to influence patients’ wellbeing and feeling of security. This was also because DNs booked follow-up appointments, made home visits when needed and because patients knew who to ask for when contacting the primary care unit.

*“The nurse came to my house and took my blood pressure, because I couldn’t go to the primary care centre and I couldn’t go by mobility service, I was in that bad condition. So that has been hard, not being able to go by yourself. Then the nurse came and visited me, she is really sweet.” Patient interview, 33*

DNs expressed satisfaction in being able to prioritize older individuals, to get to know them and addressing non-medical needs. After the intervention, they felt they had increased participants’ self-efficacy.

*“It feels like you have had the possibility to book follow up appointments with patients, without a medical reason really. So, in that aspect it feels like we have been useful and done good, and you can feel a satisfaction that you should meet someone again in a month, someone that you otherwise wouldn’t have followed up. So, when they come, you also take a blood pressure or something. Yet that was not the main thing, the conversations were. Have you made contact with your family since your last visit and did you go on that trip or… “ DN 3*

*“Otherwise, the visits tend to be quite superficial—the other patients you meet—because you’re only doing these selective measures. But here, you really have the chance to see the whole person.” DN 1*

*“In some way, I feel that—because I haven’t been completely passive in this, just sitting and listening and agreeing—I’ve actually tried to come up with some suggestions and ideas about what can be done and what alternatives there are. And I feel that for those who have come to me, something has actually happened.” DN 2*

Regarding holistic thinking, DNs saw GPs as better able to address psychosocial situation than before the intervention and GPs expressed feeling unburdened by being able to help individuals in frequent contact with the healthcare unit because of non-medical problems.

*“But some patients, often older ones, seeking contact often, it was a quite good way to transfer him to the nurse, who I worked closely with. And then I felt I wasn’t as involved”,* GP 2

DNs identified a need of intervention development involving helping individuals contacting the municipality if needed and to have supervision by a psychologist with geriatric competence to further address psychosocial situation.

##### Low priority and lack of time leads to ethically stress and frustration, and some participants do not receive the intervention

Some patients who felt in need of the intervention and only had a first and final visit were disappointed and felt the intervention was meaningless. They described contacting the primary care practice as usual during the intervention period without experiencing improved availability or continuity.

*“I somewhat had an expectation to have something more. Maybe I had an expectation to see my GP more, or an expectation to meet someone with cordiality, or I don’t really know what expectations I had. But I think I had some expectations. And I don’t really know, maybe my expectations were unrealistic in some way?” Patient, 34*

*“Well, it was still the same problem in the primary care practice. And I mean it, nothing has happened really. But I believe it has gotten really hard to get in contact with the primary care practice”, Patient, 23*

DNs expressed ethical stress when prioritizing intervention participants with non-medical needs ahead of others with medical ones. This resulted in some DNs to adhere to the intervention and book follow-ups for non-medical needs and some DNs to only adhere to the intervention and book follow-ups for medical needs.

*“I felt that many of the patients didn’t have great medical needs, so there was no need for them to come back for a second visit. Some of them I just called to check on. Someone had a little high blood pressure and had a new appointment. So, they are generally very good at taking care of their medications and health. What they experienced was that they lacked the continuity, the face-to face contact. That was quite striking actually”. DN 1*

*“I experience it very positive to not have had the pressure on you to squeeze in as many patients as possible. Yet, you feel, as it’s such a high pressure on the primary care centre, it feels like a luxury to do something like this when you know others don’t get appointments”. DN 3*

Moreover, DNs described frustration due to a time-consuming and arduous first visit addressing several areas of participant health, wellbeing, and support.

*“I think that it is the first visit that you have with the patients that takes most time and energy. And it is then you should come up with or understand what to work with.”DN 3*

Furthermore, they had mixed feelings about filling out ICAN discussion aid with the participant and writing a care plan. DNs described a need to further develop ICAN to fit into a Swedish context and especially felt the concept of patient workload was hard to comprehend. However, some DNs experienced the open answer questions regarding patient priorities in the ICAN discussion aid as helpful in identifying patients’ problems.

*“Yes, it’s hard to understand (ICAN discussion aid). It helps me—or maybe it doesn’t* *,* *I was about to say—I don’t really know, but it was hard to understand.” DN 2*

*“I had a woman who was booked for, well several symptoms. And what had been on the agenda in the GP visit was a badly regulated blood pressure, so that was the main focus. Then when we went through the questions, in ICAN, then more troubles appeared, things that she might not have seen as troubles. And then we talked about it, but it was important for me not to take over, even though I had action plans one has to stop oneself a little. And then it appeared she found it enormously boring to cook so she only ate sandwiches for all meals”. DN 3*

In addition, DNs expressed frustration when they identified a participant need that the participant were unable to see or without having anything to offer.

*“It has been pretty tough. The three first visits were mostly about to go through, well a normal sleeping cycle really and what you can do yourself. Then I thought we had agreed there weren’t so big problems, but that was not the case, but it was quite hard. So, it can be rather frustrating for oneself not reaching the patient. And there might not be so much more we can do even though it might unburden him a little, just to talk.” DN 1*

Furthermore. there was a mismatch between healthcare providers and patients experience of teamwork. Healthcare providers did not experience a need of improved teamwork, and thus did not alter how they worked together in the team during the intervention period. However, participants expressed a lack of it.

*“Well, you could discuss patients during lunch breaks. But for most of our mutual patients there was no need for a scheduled appointment, that was the idea in the project. But with some patients we did smaller medial adjustments and other things, and then you have contact”. DN 1*

Finally, DNs expressed a need for policy makers to prioritize the patient group and the intervention economically and structurally to be able to properly perform it. This involved enough time to see the whole patient and book follow-ups without having an increased workload or ethical stress.

*“And then it is important to protect older patients’ rights a bit more, otherwise they will suffer. It’s good that we guard them (their rights)”. DN 2*

#### Mixed methods results

In the joint interpretation participants experiencing a need of the intervention and receiving it as intended experienced feasibility and acceptability of receiving scheduled follow-ups and relational continuity to a nurse providing holistic care. However, in the joint interpretation several areas of development were identified before HLB can be tested in a future trial.

These areas regard better targeting individuals in need of the intervention and further assessing how person-centred and holistic care should be delivered, implemented and evaluated and what it should involve. From our quantitative findings we identified most individuals receiving a first and final visit. However, fidelity to other intervention components was low. A little bit more than 50% and 20% of the participants had a scheduled follow-up and a written care plan respectively and only three written contacts between GPs and DNs were identified in participants’ medical records. The qualitative findings were in convergence with these results and further complementarily expanded the understanding of the low fidelity.

First, many participants, despite high mean age and number of diagnoses and medications, did not experience a need of the intervention and did not want more than a first and final visit. Participants expressed reasons such as feeling healthy and capable of taking care of themselves, having a good psychosocial situation and already experiencing good structures of relational continuity to a GP and sometimes a DN. Instead, three groups of individuals with increased, most often non-disease specific, care needs were identified to potentially benefit from a future intervention. The first group involved individuals with physical and cognitive impairment, frailty, and with worse health after destabilising events. The second group involved individuals with deprived psychosocial situation including loneliness, worry, and poor coping strategies. The third group involved individuals with lack of existing structures of relational continuity and care coordination.

Second, the qualitative analysis identified that nurses experience ethical stress when prioritizing non-medical needs in intervention participants ahead of addressing medical needs in individuals outside of the intervention, leading to lower fidelity. In addition, to raise priority for the target groups providing holistic care for non-medical needs in future intervention development, it is important to make sure there is enough time budgeted to address all care needs and not miss out on intervention components.

Both quantitative and qualitative analyses described mixed results of the use of ICAN discussion aid. Even though ICAN discussion aid was filled out before or as part of the first visit (100%), it was seldom mentioned in medical records (12.5%). Qualitative findings were in convergence with the low level of written ICAN descriptions and expanded this understanding. Even though some healthcare providers experienced ICAN as helpful for addressing person-centred and holistic perspectives with the open-answer questions on the last page, it was hard to comprehend especially regarding the concept of workload. These findings inform a need to further adapt and test feasibility and acceptability of ICAN in the Swedish context, especially regarding the understanding of treatment burden with various stakeholders. However, with regards to the experiences of ICAN being hard to fill out, and the findings of potential target populations, it is important to assess if ICAN is the best person-centred and holistic approach to use or if there is a need to include or develop another one.

Regarding low levels of reported team communication (12.5%), qualitative findings were in convergence and further expanded this understanding identifying healthcare providers not experiencing a need to discuss non-medical issues. Furthermore, from the qualitative findings a need to assess improved communication between healthcare providers and participants when their views of problem differed, and between healthcare providers and other stakeholders were identified.

Finally, the quantitative findings showed high proportion of incorrectly filled out PROMs with the highest rate for the primary outcome. In the qualitative evaluation, the PROMs were experienced burdensome to fill out. Hence, there is a need to lower the number of PROMs to reduce participant burden and more thoroughly assess which PROMs to use as well as to assess for the included PROMs to be correctly filled out.

## Discussion

### Summary of the findings

In this pragmatic mixed methods, non-randomised pilot study, we found that the HLB intervention to improve patient capacity was feasible and acceptable if given as planned to the appropriate participants. Participants varied in age, number and types of conditions and medications. Most participants had a first and final visit and filled out ICAN discussion aid. However, fidelity in intervention delivery was varied as not all participants had a scheduled follow-up after the first visit(s), a care plan and the ICAN content and interprofessional communication were not documented in all medical records. The intervention was not associated with statistically significant improvements in the proposed outcomes. In addition, the completion rate of the PROMs was low. Participants and healthcare providers in the intervention unit experienced that *The most vulnerable patients need the intervention most*, and that *Patient needs and expectations need to be matched to the intervention*. The mixed methods analysis suggests that in a future trial, individuals with the greatest care needs, including those with frailty and physical and cognitive impairment, increased psychosocial needs, and lack of ongoing relational continuity and coordination of care, should be targeted. Furthermore, there is a need for intervention refinement to improve delivery, implementation, and evaluation of HLB before moving forward with an effectiveness trial. The ICAN needs further adaptation to the Swedish context. Further intervention refinement should involve raising priority of the intervention in the clinic by ensuring adequate resources for implementation, in order to create time for practitioners to provide holistic care in practice, to further assess and facilitating teamwork and communication between GP and DN, healthcare providers and participants, and healthcare providers and further stakeholders. In addition, a future trial needs to focus on one or two PROMs for outcome assessment to ensure quality of data collection.

### Strengths and limitations

A strength of this study is the testing of a person-centred intervention in a real-world setting aiming to improve patient capacity for individuals with multimorbidity. The mixed-methods study design enables us to both convergently and complementarily evaluate and gain more in-depth and a wider understanding of feasibility, acceptability and further need of intervention development than we would have gotten using quantitative or qualitative method alone. An additional strength is the use of thematic analysis with a purposive sampling with both participants and healthcare providers in the intervention unit to understand participant experiences and gain several perspectives on further intervention development.

This study has some important limitations related to its status as a feasibility study that make evaluation of effect size problematic, and lead to risk of bias. This study has risk of selection bias, as we used purposive sampling to invite two primary care units to participate without randomisation. In this study, recruitment strategy differed between the two units. In the intervention unit, the three participating GPs and DNs included participants, but in the control unit all GPs included participants. However, there were no significant differences between the demographics of included participants in intervention and control groups at baseline (Table 1). This study has some risk of performance bias. Because of pragmatic study design, participant and healthcare providers could not be blinded. In addition, participants in the intervention group may have received different treatments depending on which nurse they met. This study has some risk of detection and reporting bias. Neither the research nurse collecting PROMs in both units nor the researchers conducting data analysis were blinded. No pilot protocol was published, and the pilot study were registered retrospectively. However, trial design and outcome measurements were specified in the ethical application approved prior to trial start and the purpose of the study was to assess further need of development.

The study involves some limitations with regards to its mixed-methods study design. The aim of this study was to assess feasibility and acceptability, and need of further development of HLB. However, the mixed methods research question primarily focused on intervention development, not feasibility and acceptability. Nevertheless, feasibility and acceptability were analysed as part of the mixed methods question. Another limitation with mixed-methods study design is that it was planned as a convergent study, but the quantitative and qualitative research questions and data analyses could only partly be interpreted regarding convergence and divergence. The mixed methods analysis was therefore both convergent and complimentary.

The intervention development of HLB involve lacked the involvement of individuals with multimorbidity Intervention development was based on a systematic literature review of CC interventions, limiting the research team to use CC components when designing HLB. However, CC involves important components of relational continuity and teamwork requested by both individuals with multimorbidity and healthcare providers [20, 39, 40]. The initial plan in the research team was to conduct qualitative studies with individuals with multimorbidity, GPs, and DNs as part of intervention development [41–43]. However, due to a limitation of having a small team sensitive to changes [43],studies with individuals with multimorbidity and DNs could not be conducted as planned due to one team member dropping out. Hence, the development was influenced by a GP perspective involving a qualitative study with GPs [20] and because the research team was primarily made-up of practicing GPs. This perspective also influenced the choice of using ICAN discussion aid. ICAN was developed with involvement of individuals with chronic diseases and has been shown to be feasible in an American context [23]. However, a limitation of the development phase was lack of feasibility testing of ICAN discussion aid with people who would receive and deliver ICAN in a Swedish context [41–43]. Moreover, if we had involved stakeholders with more diverse perspectives, especially individuals with multimorbidity, we might have chosen a different person-centred approach as part of the intervention. In this study older individuals were included. However, including individuals with higher age might involve missing out on individuals with common mental health problems and poor socioeconomic situation being more prevalent in individuals with multimorbidity in younger ages [44, 45].

This study has some limitations regarding low fidelity discussed in the mixed methods results to the intervention which needs to be assessed before HLB can be tested in future trial.

Moreover, this study has some important limitations regarding inclusion rate and number of PROMs and incorrectly filled out PROMs. In this study we had difficulty recruiting participants in both units during the inclusion period. The GPs in the intervention unit expressed it hard to remember to include participants during short visits, so recruitment strategy was revised midway. Yet, in a future trial how to include participants must be further addressed. Another limitation of this study was the number of PROMs with risk of burdening participants. To address this limitation, one PROM was taken out in the start of the intervention period. In addition, PROMs were seen to be incorrectly filled out in a high extent, with the highest rate for the primary outcome measurement. Incorrectly filled out PROMs can have been influenced by number of PROMs and involves a risk of attrition bias. Hence, in a future trial number of PROMs needs to be decreased to reduce potential participant burden. This involves to more thoroughly address which PROMs to use, including choice of primary outcome, and to assess how to improve correctly filled out PROMs.

### Comparison with previous research

Our study reports an adapted CC intervention with an MDM approach to care to improve patient capacity in individuals with multimorbidity. To our knowledge this intervention design has not been tested elsewhere [18]. Previous RCTs of person-centred interventions to address individuals with multimorbidity involve heterogenous study designs and show mixed results and predominantly lack of effectiveness [18, 46, 47]. However, one of the most recent RCTs of a person-centred intervention used a mixed methods study design, identifying positive participant experiences in their qualitative analysis [46], as we did in ours.

In our mixed methods analysis, we identified a need to target the most vulnerable individuals, involving frailty, poor psychosocial situation and treatment burden. These conditions and situations can coexist [12, 48, 49] but also exist separately.

ICAN discussion aid has shown to be feasible in an American context, for an adult population with at least one chronic disease [23]. However, in a recent mixed-methods cluster-RCT published in December 2024, ICAN discussion aid showed no effect in a wide range of primary and secondary outcome measures for individuals with at least one chronic disease in an American setting. IIRS was not used as one of their PROMs [50]. Moreover, ICAN was seen to be poorly implemented. However, when it was used as intended it was experienced to add important perspectives and depth to the conversations both among participants and individuals delivering the intervention [50]. In our study ICAN was filled out by all participants yet with mixed experiences of using it identifying a need of further adaption and feasibility testing in a Swedish context. However, the findings from the cluster-RCT highlight a need to assess if implementing a person-centred questionnaire in a busy primary care practice is the best approach to make care more person-centred and holistic. A potential other approach to address in future intervention development is self-management support. Previous interventions have often involved self-management support [18, 46]. In a recent RCT testing self-management support showed positive effect in two secondary outcomes involving health behaviours and feasibility and acceptability in a qualitative evaluation [46]. Moreover, it is important to assess approaches for the identified target groups. Regarding individuals with frailty, the holistic assessment Comprehensive Geriatric Assessment is considered first choice [51] showing effect in reducing mortality in hospital settings [52] and reducing length of hospital stays and cost-effectiveness in an adapted version in a Swedish primary care setting [53]. Interprofessional communication between GP and DN were seen unchanged during the intervention and participants suggested involvement of municipality and relatives in a future trial. Cooperation between different professions can contribute to a better overall assessment of the patient’s situation [52]. Further team members can include an occupational therapist, physiotherapist, and counsellor. The team’s different approaches and skills can contribute knowledge about the complex care needs that the patient may have and improve person-centeredness.

## Conclusions

This mixed-methods pilot study shows that HLB was acceptable and feasible with regards to relational continuity and holistic nursing care when delivered to individuals in need. However, further development is needed to better target individuals with the highest care needs, such as those with frailty, psychosocial challenges, and complex care coordination. The study also highlights the need to refine how person-centred and holistic care should be delivered, implemented, and evaluated, especially from a people with multimorbidity’s perspective, and calls for greater stakeholder involvement in further intervention development.

## Supplementary Information

Supplementary Material 1.

Supplementary Material 2.

Supplementary Material 3.

Supplementary Material 4.

## Data Availability

The datasets used and/or analyzed during the current study are available from the corresponding author on reasonable request.

## Abbreviations

WHO: World Health Organisation
HLB: Health and Life in Balance
CC: Collaborative care
CM: Care manager
GP: General practitioner
MDM: Minimally disruptive medicine
QUAN: Quantitative
QUAL: Qualitative
MIXED: Mixed methods
DN: District nurse
IIRS: Illness intrusiveness rating scale
MTBQ: Multimorbidity Treatment Burden Questionnaire
EQ-5D-5L: EuroQol -5 Dimensions- 5L (31)
PHQ-9: The Patient Health Questionnaire
PSWQ: The Penn State Worry Questionnaire
WHODAS 2.0: WHO Disability Assessment Schedule
AUDIT: The Alcohol Use Disorders Identification Test, 12-item version
DUDIT: The Drug Use Disorders Identification Test
MADRS: Montgomery-Åsberg Depression Rating Scale
