# Supplementary material for: The Health and Life in Balance intervention to improve patient capacity for older people with multimorbidity: a pragmatic mixed methods non-randomised pilot study
Source: BMC Prim Care. 2025 Sep 8;26:279. doi: 10.1186/s12875-025-02974-z (PMC12418682; doi:10.1186/s12875-025-02974-z)
Supplement: Supplementary file 1 — Supplementary Material 1. [file 12875_2025_2974_MOESM1_ESM.pdf]

# ICAN discussion aid

## Capacity
**Are these areas of your life a source of satisfaction, burden or both?**

| Leave blank if not part of your life | **Satisfaction** | **Burden** |
|---|---|---|
| My family and friends | | |
| My work and finances | | |
| Free time, relaxation, fun | | |
| Spirituality or life purpose | | |
| Where I live | | |
| Getting out and transportation | | |
| Being active | | |
| Social media, TV or screen watching | | |
| My emotional life | | |
| My memory or attention | | |
| The food I eat | | |

**Workload**

**What are the things that your doctors or clinic have asked you to do to care for your health?**
**Do you feel that they are a help, a burden, or both?**

| Leave blank if not part of your life | **Help** | **Burden** |
|---|---|---|
| Take medications | | |
| Monitor symptoms | | |
| Manage my diet and exercise | | |
| Get enough sleep | | |
| Come in for appointments or labs | | |
| Recuce alcohol use, smoking, etc | | |
| Support services from municipality and other care providers* | | |
| Att hantera stress | | |
| Write in any others: | | |

*This point was changed from the original ICAN discussion aid: ”Insurance and support services”. The change was made because individuals visiting primary care do not nessarily have an insurance.

1. What are you doing to manage your stress?

2. Where do you find the most joy in your life?

3. What else is on your mind today?
