# Supplementary material for: The Health and Life in Balance intervention to improve patient capacity for older people with multimorbidity: a pragmatic mixed methods non-randomised pilot study
Source: BMC Prim Care. 2025 Sep 8;26:279. doi: 10.1186/s12875-025-02974-z (PMC12418682; doi:10.1186/s12875-025-02974-z)
Supplement: Supplementary file 2 — Supplementary Material 2. [file 12875_2025_2974_MOESM2_ESM.pdf]

Additional file 2.

Primary and secondary outcomes measures, and their comparison method.

**Patient-Reported Outcome Measures**	**Scale**	**Range**	**Comparison method**	**Statistical test**
Illness intrusiveness [1]	IIRS - Illness Intrusiveness Rating Scale (28,29)	13 items scored 1 - 7 to a total score of 13 (not very intrusive) to 91 (very intrusive) and three subdomain scores:<br>- *Relationship*<br>- *Intimacy*<br>- *Instrumental*	*Within group difference at 0 and 6 months*	Paired-sample, two-tailed, t-test for total score and subdomain scores
Treatment burden [2]	MTBQ - Multimorbidity Treatment Burden Questionnaire (30)	10 items scored to a total of 0 (no burden) to 100 (>22 high burden)	*Within group difference at 0 and 6 months*	Paired-sample, two-tailed, t-test
Quality of Life [2]	EQ-5D-5L - EuroQol -5 Dimensions- 5L (31)	A five-dimension index score with weights derived from the English population, total -0.59 to 1, (1 representing optimal health)	*Within group difference at 0 and 6 months*	Paired-sample, two-tailed, t-test

Depressive symptoms [2]	*PHQ-9* - The Patient Health Questionnaire (32)	9-items scored 0 to 3 to a total where <5 indicates absence of depressive disorder and ≥15 major depression	*Within group difference at 0 and 6 months*	Paired-sample, two-tailed, t-test
Worry [2]	*PSWQ* - The Penn State Worry Questionnaire (33)	16 items scored 1 to 5, 16 is the minimal level of worry and 80 the maximum	*Within group difference at 0 and 6 months*	Paired-sample, two-tailed, t-test
General health [2]	*WHODAS 2.0 - WHO Disability Assessment Schedule* (34)	12 items scored 0 to 4, 0 points represents optimal health and 48 worst health	*Within group difference at 0 and 6 months*	Paired-sample, two-tailed, t-test
Alcohol consumption [2]	AUDIT - The Alcohol Use Disorders Identification Test, 12-item version (35)	12 items scored 0 – 5 with a total ≤ 7 suggests low-risk; 8 - 14 hazardous; ≥ 15 alcohol dependence	*Within group difference at 0 and 6 months*	Paired-sample, two-tailed, t-test
Drug consumption [2]	DUDIT – The Drug Use Disorders Identification Test (36)	11 items scored 0 to 4, a total score ≥25 suggests dependence	*Within group difference at 0 and 6 months*	Paired-sample, two-tailed, t-test
**Clinical outcomes**	**Specification**	**Time of measurement**	**Comparison method**	**Statistical test**

Number of medications [2]	Medication count from electronic list	Baseline and post-intervention	*Between group difference at 0 and 6 months*	Unpaired, two-tailed t-tests
Number of visits in primary care (apart from the intervention) [2]	Visit count from medical records	Post-intervention	*Between group difference at 0 and 6 months*	Unpaired, two-tailed t-tests
Number of visits in secondary care [2]	Visit count from medical records	Post-intervention	*Between group difference at 0 and 6 months*	Unpaired, two-tailed t-tests
Number of admissions to hospitals [2]	Admission count from medical records	Post-intervention	*Between group difference at 0 and 6 months*	Unpaired, two-tailed t-tests

[1]Primary outcome; [2] Secondary outcome, *HLB* Health and life in balance, DN *District Nurse*, *GP* General Practitioner
