# Supplementary material for: The Health and Life in Balance intervention to improve patient capacity for older people with multimorbidity: a pragmatic mixed methods non-randomised pilot study
Source: BMC Prim Care. 2025 Sep 8;26:279. doi: 10.1186/s12875-025-02974-z (PMC12418682; doi:10.1186/s12875-025-02974-z)
Supplement: Supplementary file 3 — Supplementary Material 3. [file 12875_2025_2974_MOESM3_ESM.pdf]

Additional file 3.

Interview Guide for Patients that received the intervention

Those of you invited to this interview have all participated in the study *Health and Life in Balance*

Introduction of the interviewer

This interview is part of the research project and is not a medical consultation. What we talk about will not affect your care. Any questions about your health should be discussed with your health care provider.

Everything said in the interview will be recorded so that we can remember what is said. The content will be anonymized and used in a research article. No response in the article will be traceable to any specific person. The content will not be used in any way other than in this research project. If you wish to stop the interview at any point, just let us know. You do not need to explain why you want to stop. The interview will take approximately 30-60 minutes.

1. Do you have any questions before we begin the interview?
2. Let's begin the interview. Could you introduce yourself with your name and age? Where were you born? What language are you most comfortable speaking? What is your highest level of education? Who lives at home with you?
3. Tell us about your experience of being asked to participate in the study by your doctor or district nurse.

   a. Did your doctor or district nurse provide you with enough information about what

the study entailed?

b. What worked well, and what worked less well?

c. Any areas for improvement?

4. How did you experience the first meeting with the research nurse?

   a. Information about the study?

   b. The written information about the study?

   c. Filling out the assessment forms?

5. Tell us about your experience participating in *Health and Life in Balance*.

   a. How did you find the process of filling out the *My Life, My Health Care* brochure? What worked well, and what didn't? Areas for improvement?

   b. Then you had a meeting with the district nurse. How did you experience the contact with your district nurse? What did you do during the meetings? How many meetings did you have?

   c. What worked well? What didn't? Areas for improvement?

   d. How did you experience the contact with your doctor during the study? Did you meet during the study period?

   e. How did you feel about finishing the study? Did you feel you gained something from it? How did you feel about not having a continued planned contact afterward?

   f. What worked well and less well during the study? Things that could have been done differently? Areas for improvement?

   g. Would you recommend *Health and Life in Balance* to someone else? Why/Why not?

6. How did you experience the follow-up visit with the research nurse after the study ended?

a. Filling out the assessment scales?

b. Being asked to participate in this interview?

7. Do you feel that you have been affected by participating in this study? If so, how?
8. We've discussed many things. Is there anything else you'd like to add before we finish?

    a. Is there anything else you think I should have asked you?

9. How did you find this interview?
10. Thank you for your participation.

Interview Guide for District Nurses

You who have been invited to this interview have all participated in the study "Health and Life in Balance" with the aim of improving the well-being and quality of life of multi-ill patients.

Introduction of the interviewer

Everything said in this interview will be recorded so that we can remember everything that is said. The content will be anonymized, and in our future article, no specific answer can be traced to any specific person. The content will not be used in any other way. If you want to stop during the interview, just let us know. You do not need to say why you want to stop. The interview will take about 60 minutes.

1. Do you have any questions before we start the interview?
2. Then we start the interview. We begin with an introduction round where you all introduce yourselves with name, age, and job title.

3. Tell us about your experience participating in the study? a. What worked well/less well/what can be improved? b. How did it come about that you chose to participate in the study?
4. Tell us about your experience participating in the planning of the study before it started. a. What worked well/less well? b. Did you feel involved?
5. Tell us about your experience receiving information about the study. a. What worked well/less well/suggestions for improvement? b. How did you experience the start-up meeting? c. Information about "My Life My Care"?
6. How did you experience the communication from the research group during the study?
    - How did you experience Birgitta's presence at the health center?
    - How did you experience receiving reminders via Take Care?
    - How did you experience the lunches during the study?
7. How did you experience the inclusion of patients in the study?
    - How did you experience the information material in the form of scripts and patient materials?
    - What was your involvement in the inclusion of patients?
    - What worked well/less well/suggestions for improvement?
8. Would you describe a typical patient that the doctor in your team or you/you together have included in this study?
9. Which patients do you think would benefit from the HLB intervention?
10. How have you experienced the "Health and Life in Balance" intervention?
11. Would you tell us about a patient case where you followed a patient from start to finish in the intervention? Your experiences? What worked well? Less well? Improvement opportunities?

12. Would you describe how your patient contacts looked during the intervention?
13. How did you experience using the interview guide "My Life My Care"? What worked well and less well? Improvement opportunities?
14. How did it work to set goals together with the patients?
    - What kind of goals did you set?
15. How did your planning and scheduled follow-ups look?
    - What did you discuss during the follow-ups?
    - Did you plan from time to time or set times from the beginning?
    - Did you meet/hear on the phone/other?
16. Would you describe how the cooperation with the doctors in your teams has looked? Have you done anything differently?
    - What worked well, less well, improvement opportunities?
17. How did you experience the final visit with the patient?
    - For the patients? For you? Did you have any plan going forward when you finished? Did you have any feedback with the doctors in your teams after the end?
18. Has the intervention been able to help you in your daily work?
    - Why/why not?
19. Would you like to introduce "Health and Life in Balance" at your health center? a. Why/why not? b. Improvement opportunities? c. What would you need to maintain this way of working at your health center?
20. Anything else you would like to add?

Interview Guide for General Practitioners

You who have been invited to this interview have all participated in including patients in and conducting the study "Health and Life in Balance" with the aim of improving the well-being and quality of life of multi-ill patients.

Introduction of the interviewer

Everything said in this interview will be recorded so that we can remember everything that is said. The content will be anonymized, and in our future article, no specific answer can be traced to any specific person. The content will not be used in any other way. If you want to stop during the interview, just let us know. You do not need to say why you want to stop.

The interview will take about 60 minutes.

1. Do you have any questions before we start the interview?
2. Then we start the interview. We begin with an introduction round where you all introduce yourselves with name, age, and job title.
3. Tell us about your experience participating in the study? a. What worked well/less well/what can be improved? b. How did it come about that you chose to participate in the study?
4. How did you experience the communication from the research group during the study?

    - How did you experience Birgitta's presence at the health center?
    - How did you experience receiving reminders via Take Care?

- How did you experience the lunches during the study?

5. Tell us about your experience participating in the planning of the study before it started. a. What worked well/less well? b. Did you feel involved?
6. Tell us about your experience receiving information about the study. a. What worked well/less well/suggestions for improvement? b. How did you experience the start-up meeting?
7. How did you experience including patients in the study? a. How did you experience the information material in the form of scripts and patient materials? What worked well/less well? b. How did you experience identifying patients to ask? Opportunities/Difficulties? c. How did you experience asking patients? d. What worked well/less well/suggestions for improvement?
8. Would you describe a typical patient that you have included in this study?
9. Would you describe your thoughts on how you have included the patients that you have?
10. Which patients do you think would benefit from the HLB intervention?
11. How have you experienced the "Health and Life in Balance" intervention? a. Have you noticed any difference in the way of working? b. How has the cooperation with the district nurses in your teams looked? Improvement opportunities? c. Has the intervention been able to help you in your daily work? d. How have you experienced the patients who have participated in the intervention?
12. Would you like to introduce "Health and Life in Balance" at your health center? a. Why/why not? b. Improvement opportunities? c. What would you need to maintain this way of working at your health center?
13. Anything else you would like to add?
