# Supplementary material for: The Health and Life in Balance intervention to improve patient capacity for older people with multimorbidity: a pragmatic mixed methods non-randomised pilot study
Source: BMC Prim Care. 2025 Sep 8;26:279. doi: 10.1186/s12875-025-02974-z (PMC12418682; doi:10.1186/s12875-025-02974-z)
Supplement: Supplementary file 4 — Supplementary Material 4. [file 12875_2025_2974_MOESM4_ESM.pdf]

Additional file 4.

Number and percentage of fully entered questionnaires at baseline and post treatment

Questionnaire	Overall	HLB	TAU
IIRS n (%)	15 (28%)	3 (13%)	12 (41%)
MTBQ n (%)	39 (74%)	17 (71%)	22 (76%)
EQ-5D-5L n (%)	44 (83%)	19 (79%)	25 (86%)
PHQ-9 n (%)	35 (66%)	13 (54%)	22 (76%)
PSWQ n (%)	33 (62%)	12 (50%)	21 (72%)
WHODAS n (%)	30 (56%)	11 (46%)	19 (65%)
AUDIT n (%)	30 (56%)	10 (42%)	20 (69%)
DUDIT n (%)	32 (60%)	10 (42%)	22 (76%)

HLB Health and life in balance, *TAU* Treatment as usual, [IIRS] Illness Intrusiveness Ratings Scale; *MTBQ* Multimorbidity Treatment Burden Questionnaire; *EQ5D5L* The EuroQol 5D; *PHQ-9* The Patient Health Questionnaire; *PSWQ* The Penn State Worry Questionnaire; *WHODAS* WHO Disability Assessment Schedule; *AUDIT* the Alcohol Use Disorders Identification Test; *DUDIT* the Drug Use Disorders Identification
